# Metabolomics and Pharmacometabolomics: Advancing Precision Medicine in Drug Discovery and Development

**DOI:** 10.3390/metabo15110750

**Published:** 2025-11-18

**Authors:** Eleni V. Stolaki, Konstantina Psatha, Michalis Aivaliotis

**Affiliations:** 1 Laboratory of Biochemistry, School of Medicine, Faculty of Health Sciences, Aristotle University of Thessaloniki, GR-54124 Thessaloniki, Greece; evstolaki@auth.gr (E.V.S.); kpsatha@auth.gr (K.P.); 2 Functional Proteomics and Systems Biology (FunPATh)—Center for Interdisciplinary Research and Innovation (CIRI-AUTH), GR-57001 Thessaloniki, Greece; 3 Laboratory of Medical Biology—Medical Genetics, School of Medicine, Faculty of Health Sciences, Aristotle University of Thessaloniki, GR-54124 Thessaloniki, Greece; 4 Basic and Translational Research Unit, Special Unit for Biomedical Research and Education, School of Medicine, Aristotle University of Thessaloniki, GR-54124 Thessaloniki, Greece

**Keywords:** metabolomics, pharmacometabolomics, mass spectrometry, NMR, precision medicine, drug discovery, drug development

## Abstract

Metabolomics and pharmacometabolomics are at the forefront of precision medicine, serving as powerful tools in drug discovery and development. These approaches help address critical challenges in the field, including high clinical trial failure rates, adverse drug reactions, and interindividual variability in drug response. Comprehensive metabolome profiling enables the elucidation of disease mechanisms, identification of drug targets, optimization of therapeutic strategies, and assessment of drug safety and efficacy. It also supports more informed clinical trial design. This review highlights the pivotal role of metabolomics in advancing precision medicine and aims to broaden the perspectives of emerging scientists entering this complex field. Key analytical techniques–namely mass spectrometry and nuclear magnetic resonance spectroscopy–are discussed for their respective strengths and limitations in metabolite identification, quantitation, and structural elucidation. Additionally, analytical separation technologies such as liquid and gas chromatography, ion mobility spectrometry, capillary electrophoresis, and supercritical fluid chromatography are explored for their potential to enhance metabolome coverage, improve analytical efficiency, and reduce costs. Ongoing advancements in instrumentation and computational tools are helping to overcome major challenges in metabolomics, including metabolome complexity, data analysis and integration, and biomarker validation. These developments continue to expand the applications of metabolomics and pharmacometabolomics in both preclinical and clinical research. Ultimately, this review underscores their translational potential in facilitating drug discovery, mitigating risks in clinical trials, and shaping the future of precision medicine.

## 1. Introduction

**Figure 1:**
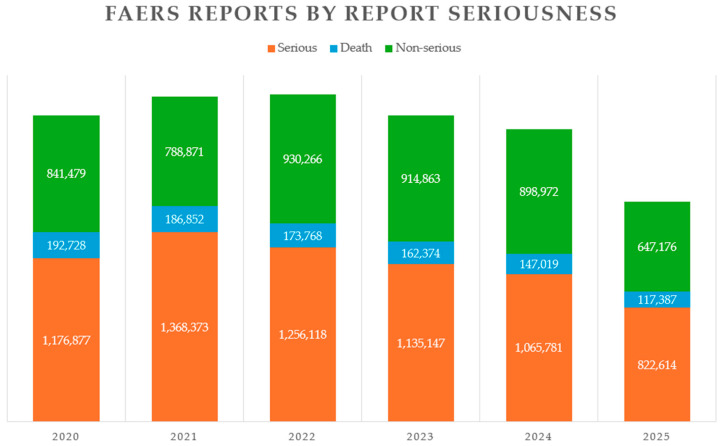
FDA Adverse Event Reporting System (FAERS) Public Dashboard reports categorized by report seriousness. The statistics cover the period from 2020 through the third quarter of 2025. Serious ADRs are depicted in orange, ADR-related deaths in blue, and non-serious ADRs in green.

More than 30% of compounds entering Phase II clinical trials fail to progress to Phase III, and nearly 60% of drugs entering Phase III do not advance further [1]. Additionally, only 25–60% of patients in clinical trials exhibit the anticipated response to treatment. These challenges are further exacerbated by adverse drug reactions (ADRs), which significantly contribute to drug-related complications, including increased morbidity and mortality [2]. Between 2020 and the third quarter of 2025, the FDA Adverse Event Reporting System (FAERS) Public Dashboard recorded a total of 9,127,716 drug-related adverse events. Of these, 54% were classified as serious adverse events, and 7.84% were associated with ADR-associated deaths (see Figure 1) [3]. Omics and systems biology technologies are emerging as powerful tools enhance the research and development of novel therapeutics. These approaches support the shift toward personalized medicine by enabling more efficient resource management and improved treatment outcomes. Metabolomics plays a critical role throughout the drug development pipeline—from refining candidate molecular targets to conducting safety assessments. It holds promise for both preclinical evaluation and clinical trials [4,5,6]. Key stages such as sample collection and preparation, separation techniques, instrumental analysis, data processing, and visualization remain complex and present numerous challenges. The physicochemical diversity of metabolites necessitates advanced expertise in analytical methods for their isolation, separation, detection, identification and quantification, along with strong capabilities in data interpretation and integration.

### 1.1. Metabolomics and Pharmacometabolomics in the Era of Precision Medicine

**Figure 2:**
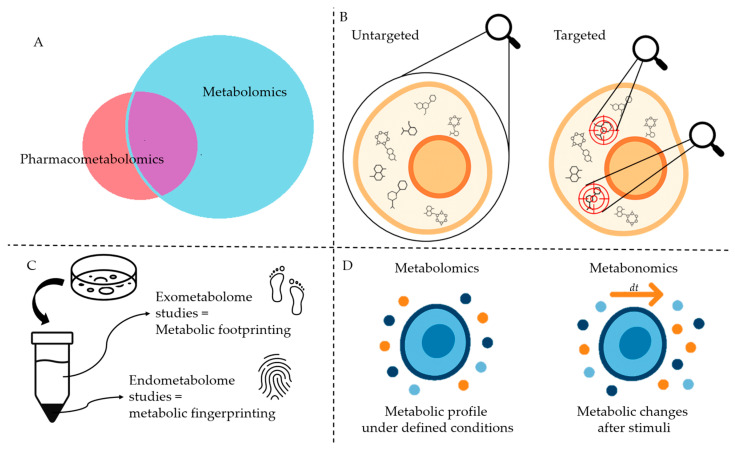
Schematic representation of terms and definitions presented in this review. (**A**). Pharmacometabolomics, a subset of Metabolomics (see Figure 4). (**B**). Targeted and untargeted metabolomics. (**C**). Metabolic fingerprinting and footprinting. (**D**). Metabolomics and metabonomics. The image was created using ChatGPT-4.5 as an assisting tool and was further refined by the authors.

Metabolomics involves the comprehensive profiling and quantification of low-molecular-weight (<1 kDa) molecules in biological fluids, cells, or tissues at a specific time and under defined conditions. A related term, metabonomics, is often described as a subset of metabolomics. It refers to the quantitative measurement of multiparametric, dynamic metabolic responses of a living system to external stimuli [7,8,9,10]. Although the terms metabolomics and metabonomics are often used interchangeably, the literature suggests a subtle distinction between them (Figure 2D). For instance, analyzing the metabolic profiles of two lymphoma subtype cell lines would be considered a metabolomics experiment, whereas examining how these profiles change in response to drug intervention would fall under metabonomics [10,11,12,13,14]. Other related terms—such as metabolic profiling or metabolic phenotyping—are also commonly used in the field.

Metabolomics and proteomics are the omics disciplines most closely linked to the phenotype, as they investigate the products, building blocks, intermediates, and by-products resulting from the regulated expression of genetic information [9,15,16,17]. Metabolome analysis complements other omics analyses by capturing information on metabolite production, fluctuation, modification, translocation, interaction, and turnover. Collectively, all omics layers contribute to a holistic understanding of an individual’s complex phenotype and underlying pathophysiology [7,17,18]. The metabolome also reflects the intricate interplay between genetic factors, nutrition and diet, gut microflora, inheritance, lifestyle, and environmental exposures. The cumulative phenotypic expression of these influences is referred to as the metabotype [19,20,21]. Studying alterations in the metabotype in response to various stimuli, such as diet, gut microbiome changes, pathological states, drug intervention, developmental stages, or environmental factors, can reveal how these influences affect the biochemical processes within the system under investigation.

**Figure 3:**
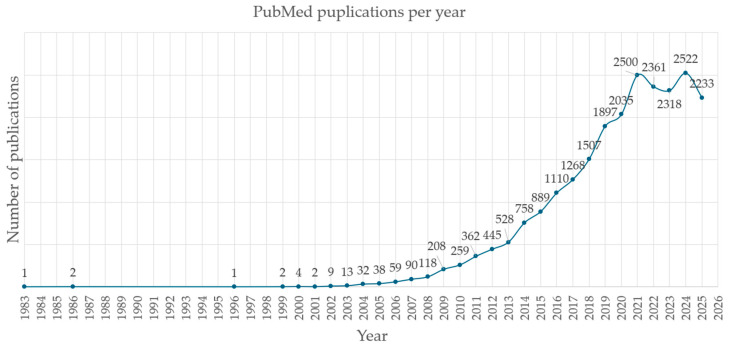
Yearly number of metabolomics-related publications indexed in PubMed (3 October 2025). Years are plotted on the horizontal axis, and the number of publications on the vertical axis. A steep increase in field-specific publications was observed during the period 2002–2021.

Metabolomics has grown rapidly over the past 25 years (Figure 3). A PubMed literature search using the Boolean query (metabolomics OR metabonomics OR pharmacometabolomics OR pharmacometabonomics OR “metabolic phenotyping” OR “metabolic profiling”) AND (NMR OR “mass spectrometry”) AND (disease OR health OR “clinical trial” OR “drug discovery”) yields 20,980 results as of 3 October 2025. Figure 3 illustrates the increasing number of articles published per year, reflecting the field’s continued growth and relevance.

**Figure 4:**
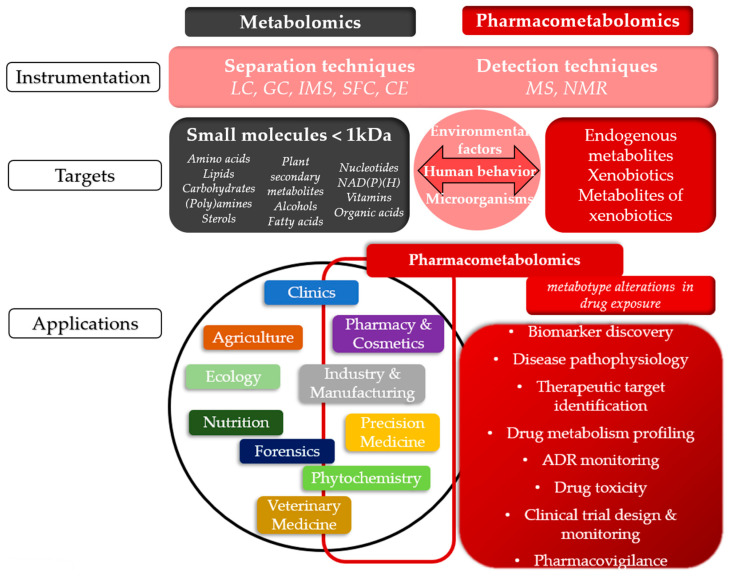
Comparative overview of Metabolomics and Pharmacometabolomics: Common Techniques, Targets, and Applications. The figure illustrates the shared and specialized aspects of metabolomics and pharmacometabolomics, highlighting their common techniques, chemical targets, and applications across various fields. The concepts shown in black and gray boxes refer to metabolomics, those in red boxes correspond to pharmacometabolomics, while the pink sections represent elements that are shared between both fields. LC: liquid chromatography, GC: gas chromatography, IMS: ion mobility spectrometry, SFC: supercritical fluid chromatography, CE: capillary electrophoresis, MS: mass spectrometry, NMR: nuclear magnetic resonance, ADR: adverse drug reaction.

**Figure 5:**
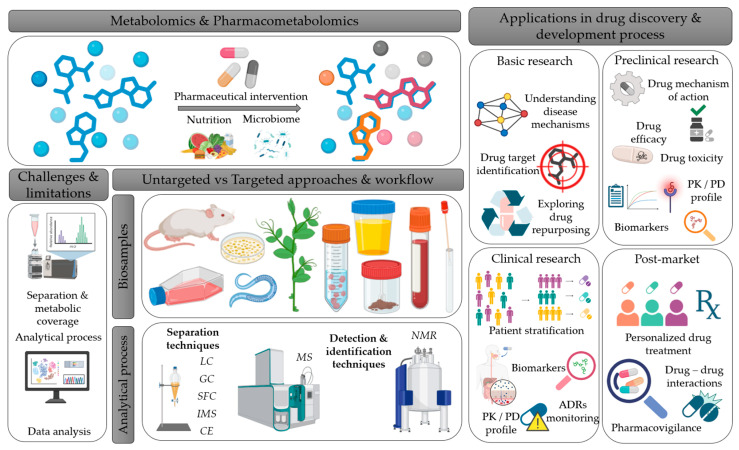
Schematic overview of Metabolomics and Pharmacometabolomics workflow, linking experimental methods to applications in drug discovery and development. The figure illustrates metabolomics and pharmacometabolomics, along with the factors influencing the metabolic profile (pharmacological treatment, diet, microbiome) and the types of biospecimens that can be analyzed. It also highlights the main analytical techniques, as well as the associated challenges and limitations. Finally, it schematically depicts the applications of metabolomics and pharmacometabolomics across the different stages of drug research and development process. This figure was created with the assistance of BioRender (https://www.biorender.com/?utm_source=moge.ai, accessed on 29 October 2025), an online illustration platform designed for creating scientific figures. LC: liquid chromatography, GC: gas chromatography, IMS: ion mobility spectrometry, SFC: supercritical fluid chromatography, CE: capillary electrophoresis, MS: mass spectrometry, NMR: nuclear magnetic resonance, PK: pharmacokinetics, PD: pharmacodynamics, ADR: adverse drug reaction.

Pharmacometabolomics, an emerging branch of metabolomics (Figure 2A), integrates metabotype data with drug exposure information to better understand and predict treatment outcomes. In other words, pharmacometabolomics leverages the pre-treatment metabolome to interpret post-treatment metabolic changes in response to drug interventions, offering insights into drug efficacy, metabolism, pharmacokinetics, and adverse drug reactions [8,21,22]. This area of research is advancing molecular and clinical pharmacology, drug discovery and development, clinical trial design, and precision medicine. As discussed later in this review, pharmacometabolomics applications underscore its potential as a prognostic tool for optimizing therapeutic strategies [8,21,22] (Figure 4 and Figure 5). Pharmacometabolomics is especially promising in addressing complex, metabolically deregulated diseases, such as cancer, cardiovascular, neuropsychiatric, autoimmune, and neurodegenerative disorders [8,21,22]. These conditions are increasingly understood as overlapping spectra of disease with shared clinical phenotypes, rather than as entirely distinct entities [22,23,24]. Moreover, the growing prevalence of these disorders—combined with the significant proportion of patients who fail to respond to otherwise effective therapies—highlights the urgent need for precise, personalized molecular insights into human diseases, as well as innovative approaches to diagnosis, treatment, and prognosis [21,22].

### 1.2. Currently Applied Metabolomics Strategies and Workflows

In metabolomics, two main strategies are employed: untargeted and targeted (Figure 2B). Untargeted metabolomics, also known as discovery metabolomics, adopts a global-scanning approach aimed at identifying patterns through metabolic fingerprinting and footprinting [7,9,25,26]. This hypothesis-generating strategy distinguishes between the intracellular metabolome (fingerprinting) and the extracellular metabolome (footprinting) (Figure 2C) [7,26,27]. Targeted metabolomics or directed metabolomics, focuses on analyzing a defined set of metabolites. This hypothesis-driven approach generally serves as a confirmatory method that complements untargeted analysis [7,9,25,26].

**Figure 6:**
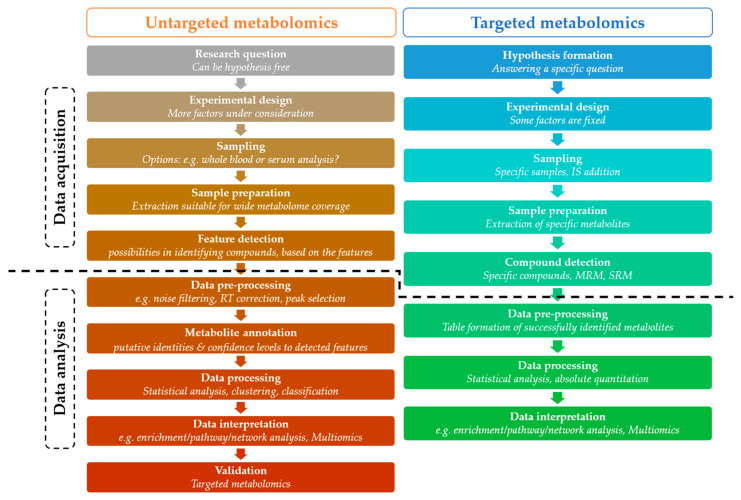
General metabolomics workflow in targeted and untargeted approaches. The figure illustrates the different modules of targeted and untargeted metabolomics analysis highlighting their basic features and dividing them into data acquisition and data analysis. IS: internal standard, RT: retention time, MRM: multiple reaction monitoring, SRM: single reaction monitoring.

A general metabolomics workflow consists of data acquisition and data analysis (Figure 6). A typical metabolomics experiment begins with the formulation of a hypothesis, i.e., defining a biological question and designing an appropriate experimental workflow to address it. The next step involves sampling, followed by sample preparation procedures, such as metabolite extraction and preparation for detection [7].

Metabolites are detected using MS and NMR spectroscopy. The acquired raw data undergo preprocessing, which in untargeted MS-based metabolomics includes noise correction, peak detection and alignment, batch effect correction, and compound identification based on spectral databases [7,28]. In NMR-based metabolomics, preprocessing primarily involves spectral alignment, baseline correction, phase adjustment, normalization, and peak deconvolution. Baseline and phase corrections are essential for eliminating instrumental artifacts, while deconvolution algorithms resolve overlapping resonances, particularly in complex biofluids [29,30]. NMR annotation relies on chemical shift reference databases, spin–spin coupling constants, and multidimensional correlation experiments such as COSY (correlation spectroscopy), TOCSY (total correlation spectroscopy), HSQC (heteronuclear single quantum correlation spectroscopy) and HMBC (heteronuclear multiple bond correlation spectroscopy) [30,31]. These 2D NMR techniques enable the elucidation of molecular connectivity and structural relationships (See Table 1).

Statistical analysis of the data can be univariate or multivariate, revealing significantly altered metabolite levels and uncovering classification or clustering patterns. The significant results obtained from data analysis can be placed in a relevant biological context through enrichment, pathway, and network analyses. Metabolomics data can also be integrated into a multi-omics framework to provide a more holistic metabolic perspective, incorporating potential changes in gene expression, epigenetic regulation or modulation and protein expression, and enzyme activities. The findings can then be validated through follow-up experiments [7,28].

Table 2 summarizes widely used bioinformatics tools, databases, and spectral libraries applied in untargeted MS-based metabolomics workflows. It is important to note that several tools serve multiple functions within the analysis pipeline. For example, MetaboAnalyst supports data preprocessing (e.g., LC-MS spectral processing), peak annotation, statistics analysis and downstream analyses such as enrichment, pathway, and network analysis.

## 2. Analytical Methodologies in Metabolomics and Pharmacometabolomics

Despite significant scientific advances in the field, there is no universally optimal platform for metabotyping. Sensitivity, resolution, throughput, and reproducibility depend on instrumentation and analytical techniques employed in each metabolome study. Selecting an appropriate platform remains a challenging process, demanding knowledge of the potential and applicability of each instrument or method’s strengths and limitations to the specific study goals and biological sample.

MS and NMR spectroscopy are powerful analytical platforms widely used for the detection, identification, and structural elucidation of small molecules in biological samples. Given the vast number of metabolites and their remarkable physiochemical diversity, coupling MS and NMR spectroscopy with separation techniques is essential [7,8,16,25,26,31]. Commonly used separation methods include GC, LC, SFC, IMS, and CE (Table 3). The choice of analytical platform and instrumentation depends on factors, such as sample type, experimental design, cost and available expertise. The following sections discuss the selected separation techniques provide an overview of MS and NMR and explore the prospects of their combination [48,49,50,51,52,53,54].

### 2.1. Biosamples and Sample Preparation

Biospecimens are highly complex analytical samples containing compounds that span a wide range of volatility, solubility, hydrophilicity, and concentration levels [55]. They comprise an astonishing diversity of metabolites with varying polarities, and average polarity depending on both the biospecimen type and the sample preparation protocol [19].

Metabolome analysis can be conducted on a plethora of biospecimens, including biofluids (such as plasma, serum, cerebrospinal fluid, urine, saliva, sweat, maternal milk, semen, and bile), as well as stool, breath, tissue, and cell cultures samples [7,9]. Cell cultures are particularly versatile, enabling simultaneous fingerprint (intracellular) and footprint (extracellular medium) profiling [56]. Sample preparation methods, such as homogenization, cell lysis, filtration, extraction, dilution, centrifugation and protein precipitation, or combination thereof, depend on the sample’s nature, complexity, and the objectives of the study [7,50,57]. Protein precipitation is a crucial step prior to analysis, as it enhances chromatographic separation and metabolite detection and identification. Protein precipitation and extraction can also be performed simultaneously by selecting the proper extraction solvent [58,59].

Between sample collection and analysis, metabolites in biospecimens are vulnerable to enzymatic degradation and chemical alterations caused bytotemperature fluctuations, exposure to oxygen, or light-induced structural changes. Quenching is the process of stabilizing and solubilizing metabolites to prevent such degradation [7,52,60]. This step is of great importance in studies of intracellular metabolome or in single-cell metabolomics, but less critical for extracellular material (i.e., plasma or urine) [7,60]. Proper storage conditions, such as keeping the samples in low temperature (freezer at −80 °C), dark, and almost full vials, are essential to avoid degradation and ensure data reliability [50,57].

The choice of extraction methods and solvents depends on the sample properties, the targeted metabolites/metabolite classes, the available or selected separation techniques and detection methods. Sample collection, acquisition, and preparation should be conducted properly to minimize losses of metabolites or potential contamination, thus, enabling metabolomic data integrity [57].

### 2.2. Separation Techniques

#### 2.2.1. Liquid Chromatography

LC is widely used in metabolomics due to the variety of available columns in length, width, and chemical characteristics of stationary phases, permitting broad metabolic coverage [52,61]. Τhe packing material of the column and the selection of the elution solvents define separation capacity. High-performance LC (HPLC) columns have internal diameters of 2.0–4.6 mm and 5–25 cm length, filled with 3–5 μm particles. Sub-2 μm particles have lately been gaining ground in metabolomics, introducing Ultra HPLC (UHPLC), which improves peak resolution, separation capacity, and metabolic coverage, while reducing the time of the analysis in comparison to HPLC [26,49,50,51,52,62]. HPLC coupled via electrospray ionization with MS (ESI-MS) is a common platform for metabotyping because it allows for the separation of compounds with very different polarities, depending on the system setup and the applied condition, such as the elution system, column selection, and instrument parameters. However, none of the current separation techniques are capable of separating efficiently all the compounds in a sample under investigation. A multidimensional approach, such as combining two (or even three) separation techniques, including Hydrophilic Interaction Liquid Chromatography (HILIC), reversed phase (RP), and/or ion pair chromatography (IPC), provides much wider metabolome coverage. RPLC is particularly effective for separating non-polar, lipophilic metabolites. The most widely used stationary phases in RPLC are non-polar, composed of silica gel modified with C8 or C18 aliphatic chains, while the mobile phase is of a higher polarity. In RPLC, non-polar or less polar compounds are retained longer by the non-polar stationary phase, while more polar compounds are eluted faster by the polar solvent system of the mobile phase [48,51,61,62,63].

On the other hand, polar aqueous compounds, such as those in urine samples, which often exhibit poor peak resolution in RPLC, are usually separated by HILIC [48,49,58]. HILIC utilizes a polar, hydrophilic stationary phase and a polar organic or aprotic mobile phase, such as mixtures of water and methanol or/and acetonitrile. Retention mechanisms involve partitioning, electrostatic interactions, and hydrogen bonding [63]. While RPLC produces narrower and more symmetric chromatograms, HILIC requires longer column equilibration times and has lower loading capacity, which can reduce sensitivity [63]. In cases where HILIC is not efficiently separating targeted metabolites, other options should be tried such as normal phase (U)HPLC, IPC or ion exchange chromatography. Usually, RHLC and HILIC are undertaken separately from one another. During the past decade, attempts were made to mechanically combine the two methodologies, including parallel separation using both columns, column switching, and serial column-coupling [58].

However, LC is not without limitations. Challenges include long runs, high solvent consumption, and the risk of artifact formation, which can lead to metabolite loss or false detection. Ion suppression due to co-eluted metabolites reduces detector response and increases background noise [26,59,64]. In addition, variability in sample preparation, extraction, chromatographic conditions, and instrument settings increases batch effect that makes the comparison of metabolomes across studies difficult [26,64].

#### 2.2.2. Gas Chromatography

In GC, the sample is vaporized upon injection and enters the instrumentation in the gas phase. The mobile phase consists of an inert gas, such as nitrogen or helium, while the stationary phase generally consists of a thin liquid film. Analytical columns in GC are placed inside a heated oven, as the heat aids to elute the compounds of different boiling points. Columns can be either packed columns or capillary columns. The use of the latter has prevailed, as they ensure improved separation performance. The length of capillary columns is noticeably greater than the length of columns used in LC. (e.g., a column in GC can be several meters long, whereas in LC it is usually between 10 and 25 cm). Physicochemical properties of the analytes, such as boiling point and polarity, determine the partitioning between the stationary and the mobile phase. The better a compound is retained in the stationary phase, the longer its elution time will be from the column, thus achieving separation [51,65].

GC is appropriate for the separation of low molecular weight (semi)volatile chemical entities. Highly polar molecules such as amino acids, sugars, and small organic acids can be analyzed by GC. Derivatization is essential to convert these compounds into their volatile derivatives but also to prevent the conversion of compounds into their stereoisomers due to high temperature (e.g., carbohydrate cyclization). Therefore, chemical processing of molecules via derivatization improves chromatographic performance, as it increases thermal stability and enhances peak shape, resolution, and intensity [51,53,66].

GC presents some advantages over LC, such as efficiency, chromatographic resolution, and high repeatability, both in retention time and response [67]. At the same time, operating and consumable costs are lower compared to LC. As for ionization techniques, GC can employ either hard techniques such as electron impact (EI), or the soft ionization technique of ESI. EI, compared to ESI, produces complex, yet reproducible spectra, allowing for more accurate identification of a compound at the MS/MS level, especially when high-resolution mass analyzers are used, such as time-of-flight (TOF) instrumentation. There are publicly available GC spectral libraries, such as NIST and Fiehnlib, in which experimentally derived spectra can be matched, as well as free and reliable deconvolution software, such as AMDIS. As a result, peak deconvolution of co-eluted compounds in GC is more accurate than in LC [67,68].

However, there are also limitations. Initially, GC-MS is restricted to the analysis of semi-volatile, volatile, and thermally stable compounds, offering limited metabolite coverage compared to LC-MS [53]. Many biological metabolites, such as amino acids, carbohydrates, organic acids, nucleotides, and lipids are not naturally volatile. Moreover, living organisms, and consequently the biomolecules that compose them, have evolved to remain stable only within a defined physiological temperature range. Furthermore, more extensive sample processing is required prior to analysis due to derivatization. The sample preparation time and the conditions required for derivatization may lead to the destabilization of metabolites and the creation of artifacts in biological samples [53,69]. In recent years, multidimensional GC×GC approaches are being explored to achieve better separation and reduce co-eluted compounds in highly complex mixtures like biosamples [53,69].

Volatile compounds are now considered a separate field, volatolomics, in which GC is the dominant separation technique before applying MS. Volatolomics finds applications in life sciences and beyond, as volatile components may originate from organisms (mammals, plants, insects), or from human or natural activity (industry, natural environment). Certain biofluids are indicative candidates for such analyses, including urine, sweat, and exhaled breath [54,65].

#### 2.2.3. Supercritical Fluid Chromatography

SFC was first reported by Klesper and colleagues in 1962, but it gained traction in the late 1980s, with the introduction of Ultra High Performance SFC, which employs sub-2 μm columns [70]. The main solvent in SFC is supercritical CO_2_ under high pressure, though combinations with co-solvents, such as ethanol and acetonitrile, enable adjustments of retention times. The main advantage of SFC is its ability to simultaneously separate polar and non-polar metabolites, thus offering wide metabolome coverage in a single run [20,48,70]. SFC is compatible with MS and can be directly coupled with a supercritical fluid extraction, providing efficient, automated extraction and separation from unprocessed biosamples, such as dried blood or serum spots, due to the high diffusivity and low viscosity of supercritical CO_2_. Moreover, SFC is ecofriendly and cost-effective, due to the use of recyclable CO_2_ instead of organic and chlorinated solvents. SFC is also applied in chiral separation, particularly in pharmaceutical studies of metabolism and toxicity [20,48,70]. While SFC shows promise in lipidomics and urine analysis [59], its application in metabolomics is limited compared to LC, due to the lack of comprehensive data libraries, including retention times and spectra, restricted stationary phase options, and challenges in quantitation [59].

#### 2.2.4. Ion Mobility Spectrometry

IMS separates ions based on their mobility in buffer gas, under an electric field, whereas the ion mobilities depend on their size, charge, and shape. Smaller and more compact ions exhibit higher mobility and thus attain higher drift velocity under the same electric field. Common types of IMS, based on ion separation, include Drift Tube IMS (DTIMS), which uses a constant low electric field, applied in a drift tube filled with a high-pressure buffer gas, such as nitrogen or helium, whilst traveling wave IMS (TWIMS) uses an oscillating electric field. Ion separation in IMS is governed by the probability of collisions with buffer gas molecules, quantified as the collisional cross section (CCS, Å^2^), which reflects the ion’s size, shape, and charge distribution [49,71,72]. IMS can be hyphenated to LC-MS, enabling separation of isobaric compounds, and decreasing noise interference, thus improving specificity and accuracy of the measurement. The LC-IMS-MS approach enables the detection of low-abundant small molecules, improving library matching and reducing false discovery rates. This occurs because the combination of two different separation techniques enhances the probability of accurate metabolite annotation, as more physicochemical properties and molecular characteristics of each compound are taken into account.

IMS is a rapid technique, permitting high-throughput separation in milliseconds, compatible with TOF and Orbitrap analyzers. Although SFC-IMS-MS has been used in a few studies until now, its potential for wider coverage of metabolome is promising. IMS coupled with LC-MS enhances metabolic tissue imaging, unveiling cellular metabolic heterogeneity and complexity. However, advanced bioinformatics tools are needed to exploit the wealth of such data [71,72,73]. Moreover, LC-IMS-MS can differentiate structural isomers, such as phase Ι drug metabolites that have undergone hydroxylation at different sites and distinguish drugs and their metabolic derivatives from endogenous isobaric metabolites [49,72]. IMS coupled to MS and Infrared Spectroscopy (IR) further enables gas-phase structural characterization of metabolites and peptides [73], while IMS alone can also shed light onto protein–drug interactions [74].

#### 2.2.5. Capillary Electrophoresis

In CE, the analyte is injected into a narrow, fused silica capillary filled with a background electrolyte in the aqueous mobile phase. Separation occurs through the application of an electric field, and analytes migrate through the capillary, in free solution, at a rate that depends on their charge, size, and shape [48,51,75,76,77]. Polar ionogenic metabolites are ideal for CE-MS analysis [77].

It should be noted that separation in both IMS and CE depends on charge, size, and shape. The main differences between the two techniques lie in the fact that in CE, the separation occurs in a liquid phase, whereas in IMS it takes place in the gas phase. The migration time of an ion through the capillary depends on its electrophoretic mobility, the length of the capillary, the applied voltage, and the electro-osmotic mobility generated by the charge on the capillary wall [48,75]. That electro-osmotic flow is responsible for the migration of neutral analytes [51]. On the other hand, in IMS, ion separation is based on their mobility in the gas phase, through collisions with the buffer gas.

CE can be coupled with MS, although it is more technically challenging to set up than LC, and is not suitable for separating hydrophobic compounds. The separation efficiency and sensitivity of CE are very high compared to chromatographic-based methods, as there is no mass transfer (partitioning) between phases [77,78]. Additionally, injection volumes in CE are in the nanoliter range, meaning that only microliter-level volumes of biosamples are required [51,77,78]. The use of aqueous solvents makes the technique more environmentally friendly compared to LC, where costly organic solvents are often used. CE analysis is enantioselective, which is important for the separation of stereoisomers [52,79]. CE is performed across different levels of biochemical analysis, such as genomics and proteomics, which may facilitate multiomics data integration in the future.

### 2.3. Instrumental Analysis for Detection and Identification of Metabolites

#### 2.3.1. Mass Spectrometry

MS is the preferred method for qualitative and quantitative metabolome profiling [61]. The molecules in the sample under investigation are converted into ionized particles (cations or anions). The ionization source can vary. The generated ions are accelerated and separated based on their mass-to-charge ratio (*m*/*z*), using electric and/or magnetic fields. The method of separation depends on the type of mass analyzer used. The separated ions reach a detector, which records their intensities and *m*/*z* values, generating the mass spectrum [16,28,51]. It is a common practice in metabolomics to acquire MS spectra of the same sample in both negative and positive ionization mode, thus enhancing metabolome coverage.

ESI, being a soft ionization method, is most widely employed in metabolomics, showing little or no in-source fragmentation. Other ionization techniques include atmospheric pressure photoionization (APPI), atmospheric-pressure chemical ionization (APCI) and matrix-assisted laser desorption ionization (MALDI). The combination of different ionization methods can expand metabolic coverage [26,61,62]. High mass accuracy is important in metabolomics, as many different metabolites with similar molecular weights may co-elute [62]. Common analyzers include Orbitrap, single or triple quadrupole and quadrupole ion trap in targeted metabolomics, and Orbitrap, Fourier Transform Ion Cyclotron Resonance (FT-ICR), TOF, and quadrupole TOF (QTOF) in untargeted metabotyping. QTRAP analyzers, which resemble a triple quadrupole, except that the third quadrupole has been replaced by an ion trap, are also utilized [16,61,64]. Samples are typically introduced into the mass spectrometer after chromatographic or electrophoretic separation [63], as direct infusion analysis often causes ion suppression, causing significant loss of signal [61]. Despite its high cost of the analysis and limitations in distinguishing isobaric compounds, MS remains a cornerstone of metabolomics [62].

**Figure 7:**
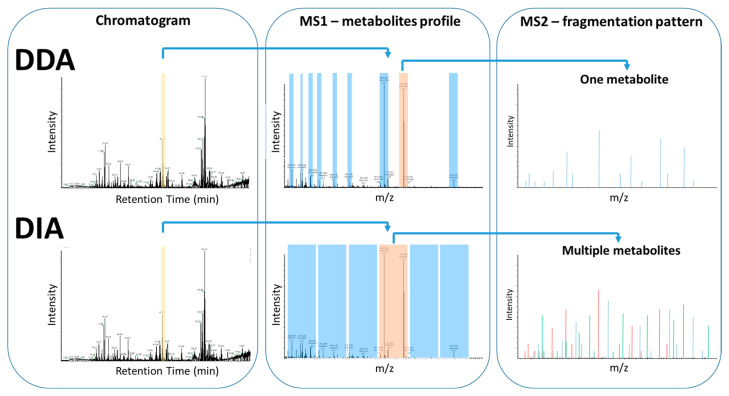
Comparison of DDA and DIA data acquisition methods. Both approaches, in most cases, acquire a MS1 scan of a mixture of molecules injected into MS, following LC separation. In the case of DDA, individual precursor ions are selected, isolated, and fragmented to acquire a corresponding MS/MS. In the case of DIA, instead of individual precursor ions, a pool of precursor ions is selected for a specific *m*/*z* window and all of them are fragmented simultaneously to acquire a consensus MS/MS.

MS spectra are not always reproducible among different instrumental platforms and also not between the same instruments with different instrumental parameters setup. Typical MS-based approaches in metabolomics apply statistical methods to identify metabolites whose concentrations are significantly different between control and studied groups. LC is typically coupled to tandem MS, where the instrument has two mass analyzers, the first measures *m*/*z* of ionized metabolites (MS1 scan), then these ions are entering a collision cell to collide with gas molecules producing ion fragments, which are then analyzed in the second mass analyzer (*m*/*z* of fragments, MS/MS) [80]. The combined MS/MS data, retention time, and precursor ion *m*/*z* enable more accurate compound identification. The precursor ion is selected either in a dependent or independent manner. In DDA approaches (data-dependent acquisition), precursor ions are automatically selected based on intensity thresholds after a full MS1 scan, whereas in data-independent acquisition (DIA) approach, fragmentation occurs to all precursor ions within a specified *m*/*z* range, regardless of intensity (Figure 7). DIA MS/MS spectra are highly complex because they contain fragment ion from multiple precursor ions, making it challenging to match precursor and fragment ions and therefore identify the metabolite [9,56,80,81]. The current application of MS/MS in state-of-the-art MS-based untargeted metabolomics approaches provides an affordable and reliable tool for metabolite identification, reducing significantly the required time and effort.

Metabolite identification framework involves the following steps: metabolite annotation, mass-based search, spectral interpretation, and spectral matching [79]. Ion annotation is the process of clustering compounds by their elution profiles. Extracted ion chromatograms (XICs) group peaks likely originating from the same compound, aiding in resolving overlapping peaks, or identifying potential isomers [79,81]. Mass-based searches deploy *m*/*z* values to create a list of putative identifications, considering adduct ions, neutral losses, and isotopes. Spectral interpretation entails in silico fragmentation predictions to match experimental spectra with a hypothetical MS/MS spectra “collection”. Findings are verified via spectral matching, where the experimental MS/MS spectra are aligned with those of reference compounds in spectral databases [55,79].

MS-based metabolomics faces several challenges, including calculating false discovery rates (FDR) unpredictable fragmentation patterns, rather low reproducibility in LC-MS/MS [55,81]. Low-abundance metabolites are often disregarded due to software limitations. An integrated feature extraction strategy in LC-MS/MS, based on classifying detected features by confidence levels, can improve metabolic coverage of low-abundance compounds. Such an approach could potentially reveal significant early-stage disease metabolic biomarkers [6,82].

#### 2.3.2. Nuclear Magnetic Resonance Spectroscopy

As noted in G. Watson’s book, NMR uses radiofrequency waves to stimulate atomic nuclei (e.g., ^1^H, ^13^C, ^15^N, ^19^F, ^31^P), for their parallel spin to alternate in antiparallel, when magnetic field is applied, providing detailed structural information. Multidimensional NMR analysis combines spectra from different nuclei, permitting the discovery of novel compounds. NMR is extensively used in pharmaceutical science and industry to quantify active drugs and their metabolites in complex mixtures like biospecimens, natural product extracts and drug formulations [51].

NMR is among the most used analytical techniques in metabolomics, due to several advantages. In NMR, sample preparation is rather simple, it is a non-destructive analysis that enables quantitation of metabolites, as signal intensity is directly correlated to metabolite concentration. As for the equipment, the system is highly automated, offering stable performance and requiring less frequent maintenance compared to mass spectrometry instruments. Considering rapid performance, automation and precise quantitation, NMR is suitable for large-scale population metabolomics studies. Generally used in untargeted analysis, NMR, unlike MS, provides high reproducibility, which is maintained even between different instruments [19]. However, NMR’s sensitivity is relatively low, typically characterizing up to 200 metabolites per scan at concentrations >1 μM, highly polar compounds and inorganic, metal ions included [31]. The latest technological achievements in NMR equipment, such as high-frequency magnets (>600 MHz) and the development of cryoprobes have increased sensitivity and resolution and decreased limit of detection [83]. The technique is applicable to semi-solid and solid samples (e.g., cells, tissues, organs, biopsies), through magic-angle spinning (MAS) NMR [84]. Moreover, NMR enables in vitro and in vivo fluxomics analyses, permitting real-time evaluation of drug response at the molecular level, considering its capacity for precise quantitation [31,83,85].

### 2.4. Quantitation in LC-MS/MS and NMR

Quantitation in metabolomics depends on the orthogonal approach used in each experiment. In targeted metabolomics, commercially available isotope-labeled internal standards (e.g., ^2^H, ^13^C, ^15^N and ^18^O) are used for validation and absolute quantitation. The limited availability and high cost of these standards confine the practicality of this approach. However, isotope-labeled internal standards serve in method development and validation, such as selectivity, limits of detection and quantitation, reproducibility, and accuracy [16,60,64]. Untargeted metabolomics relies on relative quantitation comparing spectral patterns and intensities across samples to detect differential features or distinctive marks [60,64].

NMR spectroscopy is inherently quantitative, with signal intensity proportional to the number of nuclei in each molecule. That is a salient advantage of NMR, factoring in the rapidness of the technique. qNMR (quantitative) methods include relative and absolute quantitation. Relative qNMR is widely used, enabling differential comparisons in human metabolome studies. Observed changes in metabolite levels are not assessed individually, i.e., metabolite-by-metabolite, but each metabolite is studied in correlation with others. Another qNMR application in drug discovery is detecting metabolome changes before and after treatment, thus defining pharmacological response in early stages of treatment, providing assessments of drug toxicity. Absolute qNMR is independent of the molecules in question and employs internal standards for precise concentration measurements [86,87].

### 2.5. MS and NMR Combination

While most metabolomic studies employ either MS or NMR, combining the two techniques holds great promise, offering complementary strengths. MS and NMR synergy widens metabolome coverage, improves measurement accuracy, enabling monitoring of low-abundant metabolites, all with time- and cost-effectiveness. MS provides compound identification based on accurate mass determination and molecular fragmentation pattern, while NMR enables precise quantitation. Moreover, the latter enables structural elucidation of unidentified metabolites, using chemical shifts and coupling constants, and information on molecular dynamics. This combination is popular in natural product-based drug discovery studies, allowing the characterization of unknown metabolites. These metabolites are found either in the extracts of the primary material, or as xenobiotic metabolites within the biological system in which their effects are being investigated.

However, combining MS and NMR data can be challenging because of the fundamental differences in data acquisition that are based on different physicochemical properties of the compounds. Chemical derivatization with agents, such as ^15^N-cholamine to tag specific functional groups has been used to bridge this gap [88,89]. ^15^N-cholaminecarries both an NMR-detectable isotope and permanent charge that increases sensitivity of its MS detection. This approach could find applications in biomarker discovery and identification of unknown endogenous or xenobiotic metabolites [90]. Despite these efforts, the unification of MS and NMR spectral databases and the development of analytical and computational tools, such as NMR/MS translator and SUMMIT MS/NMR, are needed to allow and simplify data integration and application [89] (Table 4). Another factor that limits the widespread application of combined MS and NMR approaches is the high cost of acquisition and maintenance of each instrumentation and their consumables, as well as the fact that it is uncommon for a single laboratory to specialize in both techniques simultaneously.

## 3. Metabolomic Applications in Drug Discovery and Development

In the era of precision, phenomic medicine, systems biology, and systems pharmacology, omics technologies are pivotal for deciphering the molecular basis of diseases. Among them, metabolomics holds particular for diagnosing and monitoring diseases, assessing disease progression, predicting onset or outcomes, and guiding the design of therapeutic strategies and interventions. By comparing metabolites levels and identifying metabolic signatures between diseased and healthy individuals, metabolomics can reveal potential disease biomarkers in various biospecimens. These biomarkers offer diagnostic, prognostic, monitoring, and mechanistic insights. Metabolomics approaches have been applied across a wide range of diseases, including diabetes, obesity, cardiovascular diseases, neurodegenerative disorders, and cancer [91,92,93,94,95,96,97,98]. Moreover, emerging areas, such as exposome research, longevity studies, extracellular vehicle research, and pharmacometabolomics are gaining increasing attention. The exposome encompasses all environmental exposures an individual experiences throughout their life; consequently, pharmaceutical interventions are also considered forms of external chemical exposure [5,6,13,17,18,71,88,99,100]. For example, metabolic reprogramming, a hallmark of cancer, differs between cancer types and among individuals. Metabolomics can guide the development of more effective metabolism-based anticancer therapies. In addition, metabolic reprogramming plays a role in modulating tumor growth [101,102], immune evasion, and metastasis [103,104], further highlighting its therapeutic relevance [17,18,105].

Metabotype information, when integrated with other omics data (genomics, transcriptomics, proteomics) and clinical data, offers a comprehensive view of drug exposure effects. This integration helps clarify mechanisms underlying disease pathophysiology, heterogeneity, and interindividual variability in pharmacotherapy outcomes, as well as pharmacokinetics and pharmacodynamic (PK/PD) properties [2,4,6,9,15,16,18,22,24,106]. Importantly, metabolomics reflects an individual’s physiology, lifestyle, and environmental exposures, making it a valuable tool in data interpretation [4,15]. Comparative analyses of pre- and post- treatment metabotypes provide molecular-level insights into drug mechanisms of action, impacted metabolic pathways, and efficacy networks [6,15,22,24,106,107]. In addition, off-target drug identification through multi-omics data integration (e.g., structural proteomics, transcriptomics) enhances our understanding of drug mechanisms and potential adverse reactions [108].

Pharmacometabolomics can support toxicology by elucidating toxicity mechanisms within complex metabolic networks and identifying the metabolic pathways affected by drug exposure. Endogenous metabolites provide baseline information about an individual’s physiological state, serving as a foundation for predicting drug response and treatment outcomes. On the other hand, exogenous metabolomics-which includes xenobiotics, such as environmental pollutants (air, water), nutrients, and pharmateuticals—helps map the metabolic fate of a drug molecule. This can reveal the formation of toxic metabolites and chemically define the causes of toxicity or interindividual variability in treatment response. This approach is particularly valuable for clarifying chronic, acute, and organ-specific toxicities, especially when integrated with pharmacogenomic data [2,5,15,106,107,109,110].

The microbiome can also have an impact on disease and drug response, affecting PK/PD properties of several drugs, such as metformin, antibiotics, antihypertensives and anticancer immunotherapeutics [17,100]. Metabolomics, by incorporating microbiome data, can assess its impact on drug treatment.

Pharmacometabolomics application is vital in preclinical and clinical phase of drug discovery and development. Pre- and post-dose comparative analyses provide drug response prediction information and drug effect assessment. For example, pre-dose biofluid samples in clinical trials, compared with post-dose samples, and combined with clinical data, may reveal predictive biomarkers for toxicity or efficacy. Short term post-dose biofluid samples are used for pharmacokinetic studies, whereas long term post-dose biofluids can be exploited in pharmacovigilance studies [2].

In pre-clinical stages of drug discovery, pharmacometabolomic studies provide information concerning drug efficacy, safety, metabolism and pharmacokinetics [2,8,79]. The overlap of metabolites across species can be both a challenge and an advantage. For example, while it complicates the identification of specific metabolite origins in microbiome studies, it increases the reliability of animal models used in the early stages of drug research and development, for pharmacodynamic and pharmacokinetic properties, ADME (absorption, distribution, metabolism, excretion) assessments and toxicological and teratogenic evaluations. Moreover, putative biomarkers detected in animal models, can predict treatment efficacy, toxicity, disease progression, or prognosis after the intervention, contributing to prioritization of drug candidates and the exclusion of likely failures in later-phase development [2,18,55,100,110,111].

Drug resistance, particularly in cancer, is another significant area. Intratumor cell heterogeneity may result in different treatment responses, and single-cell metabolomics is a promising approach in discriminating treatment-responsive from non-responsive cells enabling personalized therapy [6,111,112,113].

Biomarkers are essential throughout drug research and development, predicting drug efficacy, toxicity, and response [99]. Preclinically detected putative predictive biomarkers allow early observation of drug properties, while their validation improves the efficacy of later-phase clinical trials [4,23,110,114]. Mass spectrometry allows high-throughput analysis of endogenous and exogenous metabolites, facilitating simultaneous assessment of toxicity, efficacy, and drug metabolites. This approach also identifies molecular targets and biochemical pathways affected by drugs, even not directly linked to them [6,101,102]. Integrating pharmacometabolomics and pharmacogenomics findings can prevent drug–drug interactions, define therapeutic windows and enable precise dose selection, based on patients’ metabolic profiles or disease stages [4,6,99,114].

Metabolomics and pharmacometabolomics are important tools in clinical trials, by directing participant selection in clinical trials, stratifying patients by disease stage and patients’ treatment evaluation status [4,99,100,114]. In addition, these approaches could mitigate bioethical concerns about clinical trials, by restraining drug testing and exposure duration to patients likely to benefit and identifying toxicity potential early. Pharmacometabolomics approaches, by reducing clinical trial duration, risk and cost of drug development, accelerate the development of safer, more effective new therapies [4,114]. Pharmacometabolomics can also be applied in drug rescue and drug repurposing studies [4,6,114]. Continued advancements in these fields will further refine their applications, benefiting both research and patient care.

As the global population ages, polypharmacy, and ADRs are responsible for approximately 5% of urgent hospital admissions [115,116]. Moreover, the study of Bouvy et al., 2015 indicated that 5% of patients experience an ADR during hospitalization [116]. Beeler et al., 2023, indicate that approximately 2.3% of hospital admissions per year are caused by ADRs [117]. According to FAERS, approximately 172,000 deaths per year were associated with ADRs between 2020 and 2024 (Figure 1). Pharmacometabolomics can intensify pharmacovigilance (Phase IV drug development), by analyzing non-invasive samples in large-scale studies, identifying patients at risk for ADRs early, and improving drug safety.

The following table (Table 5) summarizes selected studies retrieved from PubMed, demonstrating the application of metabolomics in various stages of drug discovery and development. The table reports the analytical platform used (MS or NMR), the sample type, and the specific objective of the metabolomics approach in each case, such as biomarker discovery, toxicity assessment, or drug mechanism elucidation.

## 4. Current Limitations and Challenges

A major challenge in metabolomics and pharmacometabolomics lies in the chemical complexity of metabolomes. Small molecules differ in carbon chain length, functional groups, and ligands, resulting in a wide range of physicochemical properties, such as lipophilicity and chirality [17]. These variations, combined with the large number of metabolites present in biological samples, complicate their separation, detection, and analysis.

Limitations in current separation and detection techniques include the need for specialized methods tailored to different metabolite classes (e.g., hydrophobic, hydrophilic), as well as the high costs associated instrumentation, solvents, and internal standards [26,59,61,62,63,64]. Another significant challenge in MS-based metabolomics is ionization efficiency, which depends on both the analyte’s properties and the composition of the mobile phase [140]. Internal standards are typically used to mitigate this issue.

While MS offers high sensitivity and a broad analytical range-enabling the quantification of both low- and high-abundance metabolites-low-abundance signals can be masked by more dominant ones. Errors can occur at any stage of a metabolomics experiment, including study design, sample collection and storage, sample preparation, data acquisition, analysis, and interpretation. External factors, such as compound volatility and environmental conditions (e.g., temperature, humidity) may also impact metabolite detection [7,99]. In pharmacometabolomics, physiological variables, such as gender, age, diet, gut microbiota, and lifestyle further complicate data analysis [17,18]. Additionally, temporal and tissue-specific variations, as well as intracellular compartmentalization, must be considered for accurate biological interpretation [99,141].

Batch effects represent another major limitation, especially in cross-study integration and comparison. These refer to systematic variations introduced during sample collection, storage, preparation, analysis, and are unrelated to biological variability. If not properly addressed, batch effects can distort statistical analyses and lead to misleading conclusions-a common issue in all high-throughput omics studies.

Metabolomics and pharmacometabolomics are inherently multidisciplinary fields, requiring expertise in analytical chemistry, statistics, computational biology, and biological interpretation. Metabolite annotation is challenging because many compounds are involved in multiple metabolic pathways [141]. The high throughput and heterogenous nature of the resulting data demands advanced computational tools for processing, statistical analysis, compound annotation, and biochemical interpretation (Table 2). Platforms like MetaboAnalyst and MZMine offer ‘’end-to-end” solutions, while custom programming remain essential for tailored workflows. Nonetheless, multi-omics data visualization and integration continue to present major challenges [99,100,141,142].

Compound identification remains a major challenge in untargeted metabolomics. Despite the availability of spectral databases (e.g., HMDB, MetaboLights, BMRB, Bermingham Metabolite Library) and computational tools (see Table 2), many metabolites remain unannotated after data analysis [18,99,100,141]. This highlights the ongoing need to expand publicly available datasets and to develop more advanced computational tools to improve metabolite annotation and identification.

In recent years, artificial intelligence (AI) has significantly advanced metabolite annotation by enabling automated spectral interpretation and structure prediction through machine learning (ML) and deep learning (DL) approaches. AI models trained on thousands of reference MS/MS spectra can recognize characteristic fragmentation patterns, predict in silico spectra from molecular structures, and match them against experimental data. These models also perform feature prioritization and confidence scoring, enabling researchers to focus on the most reliable annotations [143].

Tools such as SIRIUS, via its CSI:FingerID module, employ ML-based fragmentation tree computation and molecular fingerprint prediction. Additionally, the extension CANOPUS uses DLalgorithms for chemical class prediction [34]. In parallel, NetID applies network inference and probabilistic scoring to link experimental features with plausible biochemical transformations [144].

MS enables and facilitates the detection of drug products, substrates, and endogenous metabolites that are not directly linked to specific targets. However, when accounting for interindividual heterogeneity, the resulting data can be highly variable, making it challenging to distinguish specific biomarkers from non-specific ones. Furthermore, the complexity of human diseases—along with phenotypic and molecular variation between individuals—suggest that identifying metabolic panels rather than single biomarkers may yield more robust and reliable predictions [99].

Validating potential biomarkers identified in pharmacometabolomics studies on a large scale and translating them into clinical practice remains a significant challenge [2,100]. Large-scale studies require standardized protocols for biospecimen and biofluid collection, proper storage, and the establishment of biobanks, as well as a skilled workforce to conduct and interpret the data [99]. In addition, most pharmacometabolomics research is conducted in small populations, which limits its generalizability and broader clinical applicability [2,100].

## 5. Conclusions

Metabolomics provides a holistic view of biological systems, spanning from genotype to phenotype, and incorporating environmental, lifestyle, and drug-related factors. This comprehensive insight makes it a powerful tool in drug discovery and development, enabling precise interventions and advancing translational research.

Instrumental analysis is central to metabolomics, but the process is complex and requires a thorough understanding of the strengths and limitations of available techniques for metabolite separation, detection, and identification. LC-MS is widely used due to its broad metabolic coverage but is time-consuming, expensive and suffers from issues with reproducibility. Emerging techniques, such as IMS and SFC, are gaining ground due to their time- and cost-efficient nature, with added environmental benefits. MS and NMR, used either separately or in combination, are essential for metabolome analysis across all stages of drug discovery and development.

In the era of Precision Medicine, metabolomics and pharmacometabolomics can guide every step of drug discovery and development—from revealing disease mechanisms and identifying candidate therapeutic targets, to deciphering the mechanism of action of a drug, to assessing drug metabolism, and evaluating toxicity and efficacy. These strategies also enhance clinical trial design and monitoring, facilitating the development of personalized treatments. While challenges remain, ongoing advancements in the field continue to address these limitations, further expanding the potential applications of metabolomics in research and clinical practice.

## Figures and Tables

**Table 1:** Two-dimensional NMR techniques commonly used in metabolomics. Comparison of 2D NMR experiments (COSY, TOCSY, HSQC, and HMBC) based on the type of couplings they detect and the structural information they provide. These techniques are essential for metabolite identification and structural elucidation, offering complementary insights into proton–proton and proton–heteronuclear correlations.

Technique	Type of Coupling	Information Provided
COSY	^1^H–^1^H (neighboring protons, 2–3 bonds)	Reveals correlations between neighboring protons through 2–3 chemical bonds, helping identify local spin systems and define short-range proton connectivity.
TOCSY	^1^H–^1^H (entire spin system)	Displays all protons belonging to the same coupled spin system, even if not directly bonded, allowing complete mapping of proton networks in complex metabolites.
HSQC	^1^H–^13^C (one-bond coupling)	Provides direct information on hydrogen–carbon single-bond connectivity, enabling accurate assignment of carbon resonances to their attached protons.
HMBC	^1^H–^13^C (2 to 3-bond coupling)	Detects long-range H–C correlations through 2–3 bonds, useful for identifying functional groups, reconstructing molecular skeletons, and confirming substitution patterns.

Note: HSQC and HMBC experiments are not restricted to ^13^C detection; they can also be performed with other heteronuclei such as ^15^N, ^31^P, or ^19^F, depending on the molecular composition and the analytical objectives.

**Table 2:** Open access computational tools and databases commonly used in MS-based untargeted metabolomics analyses. The tools are organized in this table based on their data content and the kind of information that they can provide.

Databases and Libraries	Data Processing	Metabolite Annotation and Networking	QC, Drift and Batch Effect Correction	Multiomics Data Integration
HMDB [32]	MzMine [33]	Sirius [34]	StatTarget [35]	CytoScape [36]
MetaboLights [37]	MSdial [38]	MetFRag [39]	QC-MXP [40]	MetaboAnalyst [41]
MassBank [42]	OpenMS [43]	GNPS [44]		PaintOmics [45]
GNPS [44]	XCMS [46]			
LipidMass [47]	MetaboAnalyst [41]			

**Table 3:** Comparison of separation techniques based on phase properties and target analytes. The table summarizes key separation techniques (RPLC, HILIC, GC, SFC, IMS, CE) highlighting differences in stationary and mobile phases and compound selectivity based on polarity or ion properties.

Separation Technique		Stationary Phase	Mobile Phase	Separation Targets
Liquid chromatography (LC)	RPLC	non-polar e.g. C8 or C18 bonded silica	Gradient elution preferred. Water, organic solvents, modified solvents e.g. with formic acid	non-polar, lipophilic compounds to moderate polar compounds
	HILIC	polar Zwitterionic, bare silica, diol, amino, amide, etc.	Gradient elution preferred. Water, organic polar or aprotic solvents, modified solvents e.g. with formic acid	polar compounds
Supercritical Fluid Chromatography (SFC)		variety of polarity	supercritical CO_2_ and co-solvents, such as EtOH or ACN	polar and non-polar compounds simultaneously
Gas chromatography (GC)		capillary (mostly) or packed columns, in heated oven	buffer gas N_2_ or He	(semi)volatile and small polar compounds
Ion Mobility Spectrometry (IMS)		Not applicable. Separation on gas phase, based on ion mobility and collisions with the buffer gas molecules	buffer gas N_2_ or He	independent of polarity
Capillary Electrophoresis (CE)		fused silica capillary columns	aqueous, containing electrolytes	polar ionogenic compounds

**Table 4:** NMR and MS comparison. The table summarizes the basic differences between the two common techniques used in metabolomics data acquisition.

NMR Spectroscopy		MS
Higher	Reproducibility	Lower
Low: >1 μΜ	Sensitivity	High: fM
Faster: highly automated	Duration of analysis	Slower: separation methods and ionization methods (+/−)
Minimal	Sample preparation	Demanding separation
Nondestructive	Sample recovery	Destructive, small amount of sample
Absolute quantitation: Signal intensity linearly proportional to the number of nuclei	Quantitative analysis	Relative quantitation Ionization efficiency? Isotopically labeled reference standards
Direct analysis of semi-solid samples (cells, tissue, biopsies) in vivo	Tissue samples	Extraction needed Some MALDI-TOF approaches
Hundreds of metabolites	Number of detectable metabolites per run	Thousands of metabolites
Little effect of history on data	History interference	Column and MS instrument performance can be affected by previous use
Instrumentation expensive but minimum conservation requirements and per sample cost relatively low	Cost	Instrumentation cost depending on the different models available on the market. Expensive: separation solvents and isotopically labeled reference standards

Table Abbreviations: NMR: Nuclear Magnetic Resonance, MS: Mass Spectrometry, MALDI-TOF: Matrix-Assisted Laser Desorption Ionization-Time of Flight.

**Table 5:** Selected publications from PubMed concerning metabolomics applications in drug discovery and development. The table lists the applications, whereas it specifies the disease or condition to which they are applied. The table also indicates whether MS or NMR was employed as the analytical platform and provides corresponding literature references.

No	Application	Disease or Condition	Sample Type	Instrumentation	Bibliography
1	Understanding disease mechanisms Drug target identification	hepatocellular carcinoma	serum, liver tissue, stool samples	MS	[91]
2	Patient stratification	obesity, cardiovascular and ocular diseases	serum	NMR	[92]
3	Drug mechanism of action	Alzheimer’s disease	model cell line	NMR	[93]
4	Biomarker discovery	prostate cancer	tissue and model cell line	MS	[94]
5	Drug efficacy Drug toxicity	heart failure	serum	NMR	[95]
6	Drug efficacy Biomarker discovery	nonvalvular atrial fibrillation	plasma	MS	[96]
7	Understanding disease mechanisms Biomarker discovery	Parkinson’s disease	plasma	MS	[97]
8	Drug–drug interactions (drug-radiotherapy) Drug efficacy	non-small cell lung cancer	human plasma and cell lines/mouse tumor fluids and serum	MS	[98]
9	Understanding disease mechanisms Biomarker discovery	food allergy and asthma	serum	MS	[118]
10	Understanding disease mechanisms Biomarker discovery	multiple myeloma	serum	MS	[119]
11	Patient Stratification Potential personalized drug treatment	autism spectrum disorder	plasma	MS	[120]
12	Drug mechanism of action Drug-protein interaction Drug target identification	natural products as pharmaceutical candidates	cyanobacteria	MS and NMR	[121]
13	Drug toxicity Pharmacovigilance	brivanib-induced hypertension	plasma	MS	[122]
14	Drug mechanism of action Drug target identification	hyperlipidemia	hamster liver tissue	MS	[123]
15	Drug metabolism PK profiling Drug efficacy	renal cell carcinoma	mouse model serum and urine & bile	MS	[124]
16	PK profiling Drug efficacy Drug toxicity	pharmaceutical candidates evaluation	nonhuman primates serum	MS	[125]
17	Drug–drug interactions Drug efficacy ADRs monitoring	healthy subjects under antipsychotic treatment	serum	MS	[126]
18	Drug repurposing Drug–drug interactions (synergy) Drug efficacy	Type-2 diabetes mellitus	serum	MS	[127]
19	Drug toxicity	sunitinib-induced hepatotoxicity	Serum, liver, cecum content, duodenum, jejunum, and ileum	MS	[128]
20	Understanding disease mechanism ADR monitoring	drug resistant epilepsy	serum	NMR	[129]
21	Drug toxicity Drug–drug interactions Biomarker discovery (drug use-related)	cisplatin-induced acute kidney injury	rat model serum and urine	MS	[130]
22	Drug metabolism PK profiling	treatment with masitinib	human liver microsomes	MS	[131]
23	Drug toxicity Pharmacovigilance	acetaminophen-induced hepatotoxicity	serum	MS	[132]
24	PK/PD profiling Drug metabolism Drug mechanism of action	gastric ulcer	plasma, feces, urine	MS	[133]
25	PK profiling Drug–drug interactions	heart transplant	plasma	MS	[134]
26	PK profiling Drug efficacy	quit smoking treatment	plasma and urine	MS	[135]
27	PK/PD profiling Drug–drug interactions	relapsed/refractory Diffuse Large B Cell Lymphoma	plasma	MS	[136]
28	ADR monitoring Patient stratification	schizophrenia	plasma	MS	[137]
29	Drug efficacy Personalized drug treatment/biomarker discovery/pharmacovigilance	schizophrenia	plasma	MS	[138]
30	PK profiling Drug metabolites	schistosomiasis treatment	serum, urine, feces (mouse) and microsomal incubation (human) samples	MS	[139]

## Data Availability

No new data were created or analyzed in this study. Data sharing is not applicable to this article.
